# Effect of dataset selection on the topological interpretation of protein interaction networks

**DOI:** 10.1186/1471-2164-6-131

**Published:** 2005-09-20

**Authors:** Luke Hakes, David L Robertson, Stephen G Oliver

**Affiliations:** 1 Faculty of Life Sciences, The University of Manchester, Manchester, UK

## Abstract

**Background:**

Studies of the yeast protein interaction network have revealed distinct correlations between the connectivity of individual proteins within the network and the average connectivity of their neighbours. Although a number of biological mechanisms have been proposed to account for these findings, the significance and influence of the specific datasets included in these studies has not been appreciated adequately.

**Results:**

We show how the use of different interaction data sets, such as those resulting from high-throughput or small-scale studies, and different modelling methodologies for the derivation pair-wise protein interactions, can dramatically change the topology of these networks. Furthermore, we show that some of the previously reported features identified in these networks may simply be the result of experimental or methodological errors and biases.

**Conclusion:**

When performing network-based studies, it is essential to define what is meant by the term "interaction" and this must be taken into account when interpreting the topologies of the networks generated. Consideration must be given to the type of data included and appropriate controls that take into account the idiosyncrasies of the data must be selected

## Background

In recent years, there has been an unprecedented growth in both the volume and the type of experimental data available to researchers interested in elucidating the biological networks that underpin the functions of living cells. To date, the majority of available eukaryotic data comes from the yeast *Saccharomyces cerevisiae*, where a variety of different networks have been subject to investigation, including gene regulatory [1], metabolic [2-4] and protein interaction networks [5]. As the majority of cellular processes are mediated by protein-protein interactions, much attention has been focused on their study in the hope that their investigation on a "global" scale will help us to understand how a dynamically interconnected system manages to perform multiple functionally related tasks while maintaining stability against deleterious perturbations.

The recent deluge of protein interaction data generated from large-scale high-throughput systematic screens [6-9] has presented us with an opportunity to create networks consisting of thousands of interacting proteins. Analysis of the resulting networks has shown that, in common with other naturally occurring and artificial networks, protein-interaction networks display a scale-free topology [10,11] and exhibit "small-world" properties [12]. The scale-free property of these networks is thought to be of particular biological significance as it confers robustness to random node loss, allowing the network to maintain its overall integrity even when a significant number of nodes are removed [13]. The concept of network-mediated robustness appears to be reinforced by the presence of a correlation between the connectivity of neighbouring nodes within the network (a feature not observed in random networks) [14]. In the yeast protein-interaction network, the observed negative correlation between the connectivity of a protein and the average connectivity of its binding partners has been seen as a possible adaptation which allows the network to be resilient to the propagation of deleterious perturbations [14]. Recently, Pereira-Leal and co-workers showed that this correlation is valid only for the yeast protein-interaction network as a whole, and that the network formed by the proteins essential for yeast growth has its own unique topological properties, including a very high degree of connectivity (97% of the proteins form a single distinct sub-network), which they postulate may have some implications for our understanding of the network's evolution [15].

Protein interaction networks are generally described using a graph theoretical approach, in which proteins within the graph (nodes) are connected by undirected links (edges) if they are found to interact. While creating a representation of the network is relatively straight forward, deciding what should be represented is often more difficult. Typically, networks are generated using interactions derived from a plurality of different experimental types, which may include protein interactions identified in both individual small-scale studies and larger systematic genome-scale screens – such as those from yeast two-hybrid (Y2H) and affinity-purification experiments. More often than not, less thought than appropriate is given to how the interactions derived from these different systems have been, or should be, combined and the possible implications that different methodologies for achieving this might have on the outcome of analyses.

The issue of data handling is of particular importance in the study of protein interactions derived from purified protein complexes. For any given purified complex that results from a FLAG or TAP tag-based experiment, it is very unlikely that every "prey" protein identified within the complex interacts directly with the "bait" protein. Other proteins or molecules (such as RNA) present within the mixture may act as scaffolds or bridges between the protein constituents. Consequently, we are unable to determine the true topology of the complex. In order to integrate this type of data with those from other experimental sources, we must first derive a set of hypothetical pair-wise protein interactions using either a "spoke", or "matrix" model [16]. The spoke model assumes that the bait protein physically interacts with each of the prey proteins in the complex but does not acknowledge any type of association between the preys. In contrast, the matrix model assumes that any two proteins within the "complex" are connected.

Here, we investigate the effect that the choice of datasets, and modelling methodology (matrix or spoke), has on the topological properties of the yeast protein interaction network and discuss our results with respect to the notion of a negative correlation between nodes within the network (in some studies, this is referred to as an "anticorrelation"). We go on to investigate the notion of a highly connected essential sub-network and, finally, we discuss the nature of the term "interaction" and how the interpretation of that term might affect research within the field.

## Results

### Data choice

**Figure 1:**
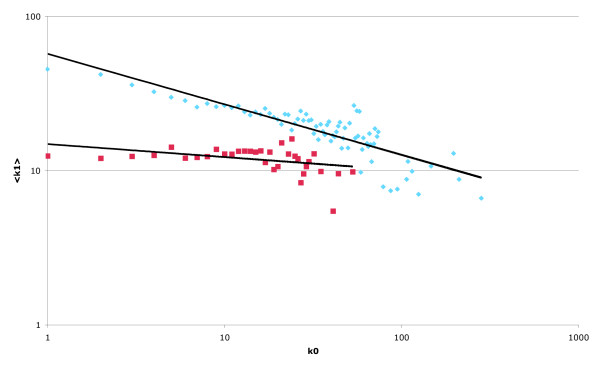
**DIP-based protein interaction network using the spoke model**. Mean connectivity of neighbouring nodes (<k_1_>) as a function of the connectivity of the central node (k_0_) displayed on a log.-log. scale for the "global" network (blue) and "essential" sub-network (red), generated from interactions extracted from the yeast subset of the DIP database. Note the difference in topology between the two networks.

The topology of the protein interaction network created using data derived from the yeast-subset of the DIP database (15,129 protein interactions involving 4,738 unique proteins) [17] reveals that the nodes within the network obey a power-law degree distribution as previously described (data not shown) [10]. Analysis of node connectivities also reveals the previously reported negative correlation between the connectivity of a central reference node (k_0_) and the mean connectivity of its neighbouring nodes (<k_1_>). The previously reported difference between the topologies of the global network and the network of essential yeast proteins is also evident, with the essential network displaying a markedly weaker negative correlation than the global network. For k_0 _≤ 60, the global network has a correlation coefficient, *r*_k0:k1 _= -0.83 and slope, αk_0_:k_1 _= -0.25; for the network of essential proteins, *r*_k0:k1 _= -0.39, α_k0:k1 _= -0.08 between log (<k_1_>) and log (k_0_) (Figure 1) [14,15].

**Figure 2:**
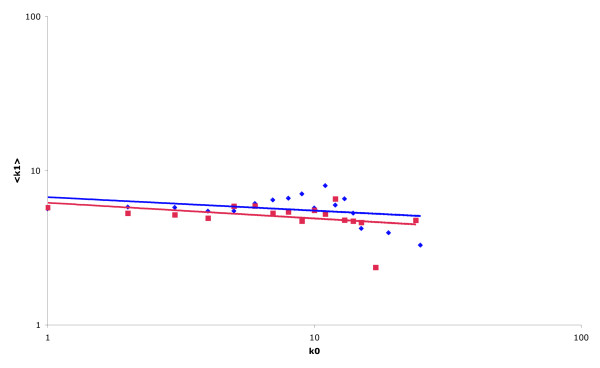
**Protein interaction network generated using the results of "small-scale studies" only**. Average value of k_1 _as a function of k_0 _displayed on a log.-log. scale for the network of non-essential proteins (red) and essential proteins (blue) generated from a dataset including only protein interactions derived from small-scale studies. Both the global and essential networks display similar topological properties with both only exhibiting a very weak negative correlation.

The yeast subset of the DIP database consists of interactions derived from a range of different studies that employed a variety of different experimental methods. It is possible that biases within one or more of these individual datasets are having a measurable effect on the outcome of the topological analysis. By performing analyses on portions of the data extracted from the database, we were able to begin to identify some of these biases. Figure 2 shows the topology of the network created from protein interactions identified in protein interaction studies (totalling 3,191 protein interactions involving 1,623 proteins) that may be characterised as "small-scale", defined as an experiment described in a published article listing no more than 100 protein-protein interactions [18]. Again, we observed the previously identified power-law degree distribution for nodes within the network (data not shown). However, in this case, the differences between the topologies of the global and essential gene networks are no longer evident, with both having similar slopes (α_k0:k1 _-0.10 global v -0.09 essential) and correlation coefficients (r_k0:k1 _-0.39 global v -0.34 essential) between log(<k_1_>) and log(k_0_). This result suggests that the significant negative correlation previously observed within the global network results mainly from data generated using high-throughput methods.

### Variation in modelling methodology

**Figure 3:**
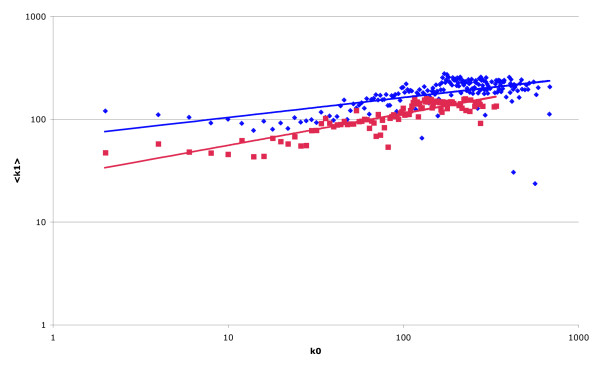
**DIP based protein interaction network using matrix model**. Average value of k_1 _as a function of k_0 _displayed on a log.-log. scale for the global network (blue) and essential network (red); generated by applying the matrix model to determine pair-wise interactions from protein complex data. For both networks, the previously reported negative correlation reverts to a positive correlation, and the previously observed difference between the topologies of the two networks appears to have been lost.

Following the dataset-dependent observations described above, the impact of applying different modelling methodologies to experimental data on protein complexes was assessed. The application of the matrix model to the protein complex data contained within the cohort used in Figure 1, and reanalysis of the resulting network, causes a dramatic shift in the topology of the network. Again, the power-law degree-distribution for nodes within the network is found. However, the previously observed negative correlation between log(<k_1_>) and log(k_0_) changes to a positive correlation for both the global and essential sub-networks, with both networks showing similar topological characteristics (Figure 3) (for K_0 _≤ 195, global network *r*_k0:k1 _= 0.68 α_k0:k1 _= 0.17, essential sub-network; *r*_k0:k1 _= 0.88, α_k0:k1 _= 0.27).

### Randomisation strategy

A second prominent finding of earlier work investigating the yeast protein interaction network is that the essential sub-network is very highly connected, with ≈ 97% of all proteins within it being connected in a single giant component [15]. The significance of this result was previously highlighted using a standard randomisation strategy, in which a number of nodes equivalent to that in the essential network were randomly selected from the global network and the connectivity of the resultant sub-network determined. To assess the validity of this finding, a "biased" randomisation strategy was employed that took into account the connectivity of the proteins within the essential sub-network. By mimicking the degree distribution of nodes within the essential network in the generated random networks, the average number of nodes encompassed within the largest connected component increased from 33% (using a standard randomisation strategy) to 88% over 1000 iterations. Although connectivity levels equal to that of the essential sub-network were not observed, levels as high as 92% connectivity were achieved.

## Discussion

In this study, we have shown how the choice of dataset and modelling methodology can profoundly affect the outcome of investigations into the topology of the yeast protein interaction network. We show that, while these variables have little effect on the apparent power-law degree distribution of nodes within the network, they can dramatically alter the correlation between the connectivities of neighbouring nodes. These results raise the question of what data should be included in these studies and, in the case of protein complex data, which of the two proposed modelling methods is the most appropriate for its incorporation? In a recent study, Bader and Hogue [16] showed that pairs identified using the spoke model were more likely to be correct (i.e. in agreement with published literature) than interactions derived using the matrix model. However, Cornell and co-workers [19] showed that there is little difference between the two modelling methods when the annotations of protein pairs found using each model were compared. This indicates that pairs derived using the matrix model are equally as meaningful (in terms of their functional annotation) as those derived using the spoke model, suggesting that either method provides a valid approach to modelling interactions. In fact, if we wish to include the "classical" hand-annotated MIPS complexes within our analyses, the matrix model becomes our only viable option, as it is the only method that allows us to define a set of pair-wise interactions for a protein complex whose topology is completely unknown.

**Figure 4:**
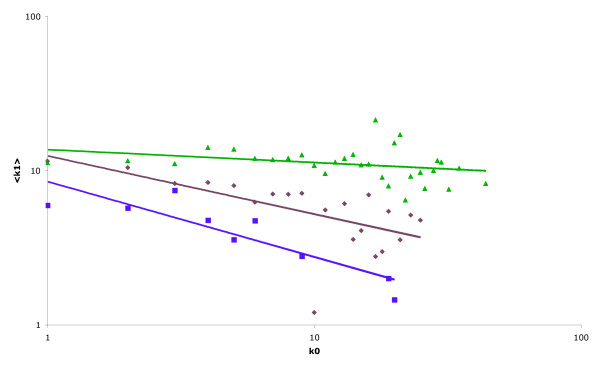
**Effect of combining data on network topology**. Average value of k_1 _as a function of k_0 _displayed on a log.-log. scale for the network composed of essential yeast genes. In each case, where the dataset includes information derived from protein complex analysis pair-wise interactions are determined using the spoke model. Green: network resulting from the incorporation of all protein interactions not classified as being obtained in "small-scale" studies. Brown: network resulting from the protein complex study performed by Ho and co-workers [7]. Purple: network resulting from Y2H interactions identified by Ito and co-workers [8]. Note that the relatively strong negative correlation present within the two networks generated using data from individual high-throughput studies (brown and purple on the graph) is significantly reduced when all available high throughput data are combined (green).

Given our results on the topology of the network, it is hard to believe that changes in the strength and polarity of the correlations observed, stem from some underlying biological process [14,15]. Rather, they are presumably the result of biases introduced either through experimental methodologies or the choice of analysis technique. As an example, data from Y2H experiments show a significant asymmetry between the connectivities of baits and preys (i.e., the average connectivity of baits with at least one interaction is almost double the same quantity measured for preys) [14]. This and other factors such as auto-activation and the presence of "sticky-proteins" [20], if not taken into account during network construction and analysis, could create the false impression of a negative correlation. In a similar way, it is obvious (from the volume of published literature) that yeast essential genes have been subject to a greater degree of investigation than non-essential genes, and are therefore liable to have more documented interacting partners. It is possible that this bias is responsible for the appearance of the topologically distinct "essential" sub-network (first identified by Pereira-Leal and co-workers [15], and shown here in Figure 1) because a more complete and "accurate" dataset is available for analysis. This is supported by the fact that this topology is only evident when multiple datasets (which, if taken individually, often show an apparent negative correlation) are combined (Figure 4). Furthermore, we have shown that, by changing the modelling methodology employed to derive pair-wise protein interactions, it is relatively easy to change the overall topology of the observed networks. Although the application of one model seems to indicate the global and essential networks have distinct topological properties (Figure 1), by using the same data and employing another (apparently equally valid [19]) model, we show that the previously observed negative correlation reverts to a positive correlation for both the global and essential networks, and the topologies (in terms of both the correlation between log(k_0_) and log (<k_1_>) and the slope of the resulting graphs) of both also appear similar (Figure 3). The difference between the topologies of the spoke and matrix model networks is probably a consequence of the models themselves. In the spoke model, bait proteins tend to have a higher degree than prey proteins; thus typical interactions within the network are between high-degree and low-degree members. Conversely, in the matrix model, all proteins found within a complex are assigned connections with all others; thus typical interactions tend to be between proteins with similar connectivities. The ease with which the overall topology of the networks is flipped, and the disparity between results given above, further highlights the degree of caution that should be exercised when attempting to draw biologically meaningful conclusions from studies of this type.

In addition to the observations made about the correlations between neighbouring nodes, we have also shown the importance of using the correct control when selecting nodes for randomization studies involving network connectivity. We found that, by simply matching the degree distribution of the nodes within the essential network in that of the randomly selected sample (composed entirely of non-essential genes), we were able to achieve very similar levels of network connectivity. This result suggests that the highly connected nature of the sub-network of essential proteins previously reported by Pereira-Leal and co-workers is primarily a consequence of the high-degree bias of its nodes, rather than a manifestation of some specific evolutionary process.

## Conclusion

We conclude that, before embarking on these network-based analyses, we must first be clear as to what we mean when we use the term "interaction". Interactions derived from direct physical studies, such as Y2H experiments, are very different from those found in synthetic genetic screens, which (in turn) are different again from "associations" between the proteins found within protein complexes. However, in several recent studies, many of these different interaction types have been lumped together as though they were equivalent and directly comparable. For instance, both Y2H data and synthetic lethal gene pairs count as 'interactions' in the GRID database [18], although the protein products of the latter rarely interact physically [21].

While graph theoretical analysis approaches have been successfully applied to a number of man-made and naturally occurring networks [22], these networks differ from the biological systems investigated in that every link between pairs of nodes within the network is of the same type and is generally independent of other factors. For example, analysis of the HTML pages that make up the content of the World Wide Web is relatively simple. In this network, both the nature of the relationships (hyperlinks) between nodes (pages) and the nodes themselves are usually homogeneous and well-defined. Therefore, meaningful and representative visualizations and quantifications of the structure of the network and its properties are possible. However, the "biological networks" we construct are not representative of the underlying system. Biological systems essentially comprise protein "machines" [23] and biological function is mediated through **associations **between proteins, either directly through physical contact, or indirectly within protein complexes, or as part of the same biological pathway. Although it is technically possible to create an abstract representation of these associations; in reality, heterogeneity and the spatial and temporal restrictions imposed upon the links mean that the resulting topology and parameters of the network need not convey biologically meaningful information.

## Methods

Network analysis was performed by extracting all machine-readable, yeast-derived protein interactions from the DIP database (release 20050605). Node connectivities and network topology were investigated using custom software written in the Perl programming language. Random networks were generated from a pool of non-essential proteins only. Construction of the random network continued until the appropriate number of proteins had been selected, whose degree distribution within the sample was similar to that actually observed in the essential network. This was done using an algorithm that created a sample of nodes that, at each level of connectivity, matched as closely as possible (data-permitting) the observed node numbers in the essential network. In instances where an exact match was not possible, another node with a degree within the same range of the desired node was selected. The essential sub-network is defined by taking into consideration only interactions between essential genes, as defined by the *Saccharomyces *Gene Deletion Project [24]. Correlations between variables were determined by computing the Pearson's correlation coefficient, *r*. We also report the slope, α, of a linear fit to the data.

## Authors' contributions

LH carried out all the analyses and wrote all the custom algorithms, with advice and help from DR, and involving discussions between all authors. LH wrote the initial draft of the manuscript, which was revised by DR and SGO (who conceived of the study). All authors read and approved the final manuscript.
